# Cytotoxic Effects of Chlorophyllides in Ethanol Crude Extracts from Plant Leaves

**DOI:** 10.1155/2019/9494328

**Published:** 2019-07-15

**Authors:** Yi-Ting Wang, Chih-Hui Yang, Ting-Yu Huang, Mi‐Hsueh Tai, Ru-Han Sie, Jei-Fu Shaw

**Affiliations:** ^1^Department of Biological Science and Technology, I-Shou University, Taiwan; ^2^Pharmacy Department of E-Da Hospital, Taiwan; ^3^Taiwan Instrument Research Institute, National Applied Research Laboratories, Taiwan

## Abstract

Chlorophyllide (chlide) is a natural catabolic product of chlorophyll (Chl), produced through the activity of chlorophyllase (chlase). The growth inhibitory and antioxidant effects of chlide from different plant leaf extracts have not been reported. The aim of this study is to demonstrate that chlide in crude extracts from leaves has the potential to exert cytotoxic effects on cancer cell lines. The potential inhibitory and antioxidant effects of chlide in crude extracts from 10 plant leaves on breast cancer cells (MCF7 and MDA-MB-231), hepatocellular carcinoma cells (Hep G2), colorectal adenocarcinoma cells (Caco2), and glioblastoma cells (U-118 MG) were studied using MTT (3-(4,5-dimethylthiazol-2-yl)-2,5-diphenyl tetrazolium bromide) and DPPH (1,1-diphenyl-2-picrylhydrazyl) assays. The results of the MTT assay showed that chlide in crude extracts from sweet potato were the most effective against all cancer cell lines tested. U-118 MG cells were the most sensitive, while Caco2 cells were the most resistant to the tested crude extracts. The cytotoxic effects of chlide and Chl in crude extracts from sweet potato and of commercial chlorophyllin (Cu-chlin), in descending order, were as follows: chlide > Chl > Cu-chlin. Notably, the IC_50_ of sweet potato in U-118 MG cells was 45.65 *μ*g/mL while those of Chl and Cu-chlin exceeded 200 *μ*g/mL. In the DPPH assay, low concentrations (100 *μ*g/mL) of chlide and Cu-chlin from crude extracts of sweet potato presented very similar radical scavenging activity to vitamin B2. The concentration of chlide was negatively correlated with DPPH activity. The current study was the first to demonstrate that chlide in crude extracts from leaves have potential cytotoxicity in cancer cell lines. Synergism between chlide and other compounds from leaf crude extracts may contribute to its cytotoxicity.

## 1. Introduction

Plants are the foundation of traditional medicines that have existed for thousands of years [1]. A number of plant extracts have been shown to possess anticancer properties, including* Annona muricata L.*,* Carica papaya*,* Colocasia gigantea*,* Annona squamosa Linn*,* Murraya koenigii L.*,* Olea europaea L.*,* Pandanus amaryllifolius Roxb.*,* Chenopodium quinoa*,* Toona sinensis*,* Myristica fragrans*,* Thermopsis rhombifolia*, and* Cannabis sativa *[2–13]. The potential anticancer activities of these plants have been associated with various bioactive compounds, including chlorophyll (Chl), pheophorbide (Pb), alkaloid, terpenoids, polysaccharides, lactones, flavonoids, carotenoids, glycosides, and cannabidiol [14–22]. Beside the possibility of anticancer functions, compounds in plant extract have been demonstrated to exert function of antioxidation, anti-inflammation, and attenuate side effects induced by chemotherapeutics [11–13].

Those bioactive factors, especially Chl and its derivatives, have demonstrated potential for the treatment of cancer [23]. Chl, the most abundant pigment on earth, is present at high levels in green leafy plants, algae, and cyanobacteria [24, 25]. The catabolic derivatives of Chl are chlorophyllide (chlide), pheophytin, Pb, and phytol [26, 27]. Studies have demonstrated that Chl can reduce the growth and proliferation of MCF-7 breast carcinoma cells [28]. Chl has also been reported to promote cell differentiation and to induce cell cycle arrest and apoptosis in HCT116 colon cancer cells [29, 30]. Hsu* et al. *(2005, 2008) showed that chlide a/b and Pb a/b reduced hydrogen peroxide-induced strand breaks and oxidative damage, and aflatoxin B1-DNA adducts formation in hepatoma cells [31, 32]. Additionally, Guo* et al*. (2011) demonstrated that chlide decreased the levels of hepatitis B virus without affecting cell viability and viral gene products in tetracycline-inducible HBV-expressing HepDE19 cells [33]. In human lymphoid leukemia molt 4B cells, pb a and phytol were able to induce programmed cell death [34, 35]. Phytol could also reduce inflammation by inhibiting neutrophil migration, reducing the levels of IL-1*β* (interleukin 1-*β*), TNF-*α* (tumour necrosis factor-*α*), and oxidative stress [36]. Pb a, in photodynamic therapy, was found to increase the levels of cytosolic cytochrome c and has also been tested against human pancreatic cancer cells (Panc-1, Capan-1, and HA-hpc2), hepatocellular carcinoma cells (Hep 3B), uterine sarcoma cells, human uterine carcinoma cells, and Jurkat leukemia cells [37–39]. Pb has also been shown to decrease the levels of procaspase-3 and -9 in Hep3B, Hep G2, and human uterine sarcoma MES-SA cells [37, 39].

Extensive studies have been performed with copper chlorophyllin (Cu-chlin). Cu-chlin, a semisynthetic, Cu-coupled, and water-soluble derivative of Chl, has been shown to significantly decrease the growth of mutagen-induced cancer cells [40–42].* In vitro* and* in vivo *studies have suggested that Cu-chlin possesses antigenotoxic functions against compounds present in cooked meat, including N-nitroso compounds and fungal toxins, aflatoxin B1 (AFB1), and dibenzo[d,e,f,p]chrysene (DBC) [32, 43–45]. The regulation of cancer growth by Cu-chlin seems to involve the deactivation of key signal transduction pathways, including the nuclear factor kappa B, Wnt/b-catenin, phosphatidylinositol-3-kinase/Akt, and expressed E-cadherin and alkaline phosphatase pathways [46, 47].

The amount of Chl degraded globally each year is estimated to exceed 1000 million tons, and this is mostly derived from agriculture and food processing waste. Except for the edible parts of vegetables and fruits, most Chl from low-value agricultural waste can only be degraded naturally. By using low-value agricultural waste as sources to collect Chl, the cost of extraction can be reduced and maximum value of agriculture waste can be reached. Therefore, agricultural waste is potentially useful in the biomedical industry as a high-value nutraceutical and pharmaceutical material. To the best of our knowledge, the problems of stability and levels of chlide leads to no comparative studies assessing the effects or functions of chlide have been conducted. In our previous studies, we cloned and expressed recombinant chlase from* Brassica oleracea*,* Chlamydomonas reinhardtii,* and* Cyanobacterium cyanothece *sp. ATCC 51142 to hydrolyze Chl into chlide and phytol* in vitro *[48–51]. We also developed a APTES-coated MIONP-immobilized recombinant* C. reinhardtii* chlase (CrCLH), which can be used repeatedly to reduce the costs of chlide production [51]. In the present study, the antitumor effects of chlase-treated ethanol extracts from 10 plant leaves were investigated. Ethanol crude extracts from sweet potato leaves were found to be most effective against the cancer cell lines tested.

## 2. Materials and Methods

### 2.1. Preparation of Leaf Crude Extracts and Chl Extraction

Plant leaves were purchased from a local market or famer in Kaohsiung, Taiwan. The leaves of guava, sweet potato, lemon, banana, Chinese toona, logan, wax apple, mango, caimito, and cocoa were used to extract Chl. Ten-grams (wet weight) of leaves was washed, dried, and ground into powder with a pestle and mortar. Leaf mixtures were then frozen in liquid nitrogen and stored at -80°C in a deep freezer. Chl was extracted by immersing leaves in ethanol solvent (HPLC grade) for 48 h. Ethanol crude extracts from leaves were centrifuged at 1500 g for 5 min and keep at -20°C for further experiments. To measure the concentrations of Chl a/b, crude extracts were passed through a 0.22 *μ*m filter and the absorbance was measured at 665 and 649 nm, which are the major absorption peaks of Chl a and b, respectively. Chl a and b contents of the leaf were converted using previously calculated equations [52–54]. Chl in crude extracts were treated with chlase to generated chlide and then lyophilized in order to measure the weight. The estimated concentrations of Chl a and Chl b in crude extracts were calculated according to the following equation: Chl a (*μ*g/mL) = 13.7*∗*A665-5.76*∗*A649, Chl b (*μ*g/mL) = 25.8*∗*A649-7.6*∗*A665 [52–54].

### 2.2. Preparation of Chlase-Treated Ethanol Crude Extracts

*C. reinhardtii* chlase (CrCLH) was produced as described previously [48, 50]. Recombinant CrCLH was expressed, purified, and then lyophilized. The reaction mixture contained 0.5 mg of recombinant CrCLH, 650 *μ*L of the reaction buffer (100 mM sodium phosphate, pH 7.4, and 0.24% Triton X-100), and 0.1 ml of Chl in crude extracts from leaves (100 mM). The reaction mixture was incubated at 37°C for 30 min in a shaking water bath. The enzymatic reaction was stopped by adding 4, 6, and 1 mL of ethanol, hexane, and 10 mM KOH, respectively. The reaction mixture was vortexed vigorously and centrifuged at 4000 rpm for 10 min to separate the two phases. The upper layer contained the untreated Chl a/b; the bottom layer was chlase-treated crude extract comprising chlide a/b. The chlase-treated crude extracts containing chlide a/b mixtures were then concentrated and the solvent was removed by evaporation under reduced pressure at 40°C on a rotary evaporator (IKA-Werke, Germany). The concentrated crude extracts were processed by lyophilization, weighed, and stored at -80°C for further experiments. The chlide standard was purchased from DHI lab products (DENMARK).

### 2.3. Cell Cultures, Chemical Treatments, and Morphological Observations

Five eukaryotic cell lines were used to assess cytotoxicity in* in vitro* assays: human fibroblast cells (NIH/3T3), human breast cancer cell lines (MCF7 and MDA-MB-231), hepatocellular carcinoma cells (Hep G2), colorectal adenocarcinoma cells (Coca2), and glioblastoma cells (U-118 MG) were purchased from the American Type Culture Collection (ATCC) (Manassas, USA). Cells were cultured in were DMEM (Dulbecco's modified eagle medium) supplemented with 10% fetal bovine serum (FBS), Eagle's Minimum Essential Medium (EMEM), with 10% FBS and 0.01 mg/mL insulin, Leibovitz's L-15 Medium (L15) with 10% FBS, EMEM with 10% FBS, EMEM with 20% FBS, and DMEM with 10% FBS. The cells were maintained at 37°C under a humidified atmosphere of 5% CO_2_, except for MDA-MB231. The cells were treated with increasing concentrations of crude extracts (50, 80, 100, 150, and 200 *μ*g/mL) and cultured in an incubator at 37°C for 48 h, and the cellular morphology was observed. Following incubation, the cells were observed under an inverted microscope.

### 2.4. High-Performance Liquid Chromatography (HPLC) Analysis of Chl Catabolites

To analyze Chl and chlide in crude extracts, chlase-treated crude extracts were analyzed using HPLC as described previously [55]. Since the provision of commercial standards was limited, it was not possible to identify all peaks in all ethanol crude extracts by HPLC. Herein, the standards used in this study, including Chl a, Chl b, chlide a, and chlide b, were selected based on our previous studies [56]. HPLC results were obtained using mobile phases consisting of ethyl acetate/methanol/H_2_O_2_ = 44:50:6. Samples were quantified based on the retention times and UV spectra compared with the standards. Chl and chlide were detected at a wavelength of 667 nm and identified by absorption spectra, peak ratios, and comigration with authentic standards [57].

### 2.5. Colorimetric MTT Viability Assay in Cancer Cell Lines

Cell viability was examined by the ability of the cells to cleave the tetrazolium salt MTT [3-(4,5-dimethylthiazol-2-yl)-2,5-diphenyl tetrazolium bromide] (Sigma Chem., St. Louis, MO) by the mitochondrial enzyme succinate dehydrogenase following a previously described procedure [58]. Cells were incubated at the temperature used to acclimatize cell lines. The background absorbance of the culture medium was subtracted from the measured absorbance. Cells (5×10^4^/well) were stimulated with different doses of crude extracts (50, 80, 100, 150, and 200 *μ*g/mL). At the end of the incubation period, 24 h after stimulation, 20 *μ*L of the MTT solution was added per well. After treatment for 24 h, supernatants were removed from the wells and 1% MTT solution was added to each well. The plates were incubated for 4 h at 37°C and the optical density was determined at 595 nm using a multiwell spectrophotometer (Multiskan, Thermo Fisher Scientific, Waltham, MA). All measurements made in the 96-well plates were performed using five technical replicates. In addition, cell viability was examined microscopically for the presence of cytopathic effects. The half-maximal inhibitory concentration (IC_50_) was defined as the concentration required to inhibit cell viability by 50%. The IC_50_ value and the standard error of the mean (SEM) were calculated using a nonlinear regression curve contained in the SigmaPlot™ statistical software. A calculated selectivity index (SI) evaluated the relationship between cytotoxicity of cancer cells and normal cells. The SI was calculated from the IC_50_ of normal NIH-3T3 versus cancer cells. Crude extract was considered to have high selectivity for cancer cells if the SI exceeded 2 [59, 60]. Values in Tables 1 and 2 were evaluated by linear regression analysis. Correlation coefficients between Chl/chlide content and cytotoxic activity were calculated by Pearson's correlation coefficient (Supplementary Table 1). The values were between +1 (black color) and −1 (red color). The absolute value of correlation coefficient ranges from 0.7 to 0.99, from 0.4 to 0.69, from 0.1 to 0.39, and from 0.01 to 0.09 which was defined as high, moderate, modest, and weak correlations.

### 2.6. Free Radical Scavenging Assay

The DPPH assay was used to evaluate the free radical scavenging of chlase-treated crude extract from sweet potato [16]. Briefly, DPPH (8 mg) was dissolved in methanol (100 mL) to obtain a stock solution of 80 *μ*g/mL. Then, 2.95 mL of the working solution was mixed with 50 *μ*L of sample. After incubation in a dark at room temperature for 20 min, the absorbance was measured at 517 nm. The DPPH scavenging effect (%) was determined using the following formula:

(1) $$\begin{array}{c} Kd\left(\;\%\;\right)=\frac{\text{Ac-(Ai-Aj)}}{\text{Ac}}*100 \end{array}$$

where Ac was the absorbance of the blank control, Ai was the absorbance in the presence of the samples, and Aj was the absorbance of the samples alone [61]. Vitamin B2 was used as a reference standard compound. The EC_50_ value, which is the concentration that can inhibit 50% of DPPH free radicals, was obtained by extrapolation from regression analysis [62].

## 3. Results

### 3.1. Content of Chl a/b in Crude Extracts from 10 Plant Leaves

Chl was extracted from 9 plant leaves, including guava, sweet potato, banana, toona, longan, wax apple, mango, caimito, and cocoa. The results are summarized in Table 1. This comparison enables the different plants to be ranked according to their extraction efficiency. Significantly more Chl a was observed in toona (9.8 mg/gDW), followed by mango (8.4 mg/gDW) and rambutan (7.8 mg/gDW). The lowest Chl a levels were present in banana (2.921 mg/gDW), sweet potato (3.481 mg/gDW), and caimito (5.218 mg/gDW). For Chl b, toona possessed the highest content (5.419 mg/gDW), followed by cocoa (4.485 mg/gDW) and mango (2.599 mg/gDW). The lowest levels of Chl b were found in sweet potato (0.996 mg/gDW), banana (1.031 mg/gDW), and caimito (1.493 mg/gDW). Of the species analyzed, leaves of rambutan, cocoa, and caimito contained the highest level of ethanol crude extracts (Table 1), at 534.35, 412.65, and 397.62 mg/gDW, respectively. The lowest weight of leaves crude extracts was obtained from sweet potato (43.175 mg/gDW), banana (47.76 mg/gDW), and wax apple (94.29 mg/gDW).

### 3.2. HPLC Analysis of Chl a/b and Chlide

**Figure 1:**
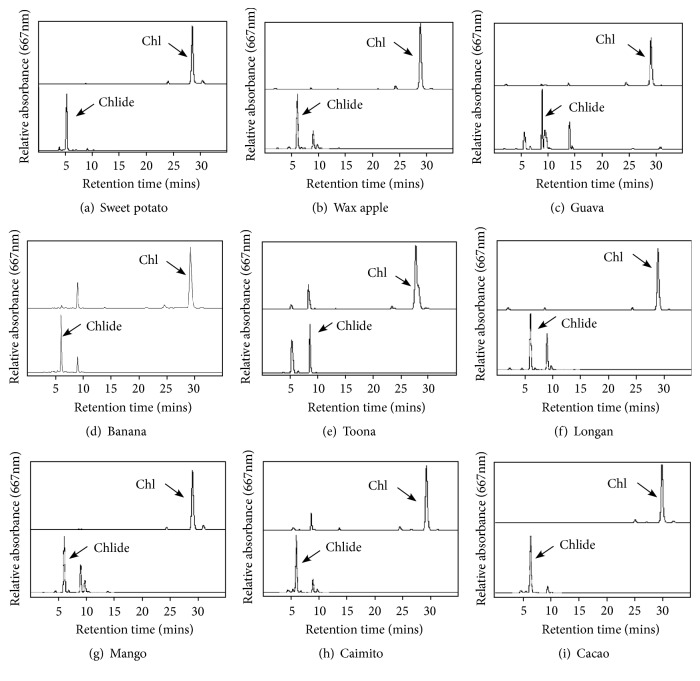
*HPLC analysis profiles of Chl and chlase-treated ethanol crude extracts from different plant species*. The products were separated by HPLC and detected at 667 nm from 0 to 80 min. Chl and chlide were detected within 30 min and 10 min.

The HPLC separation system was applied to determine the amount of Chl a/b and chlide a/b in ethanol crude extracts.  Figure 1 shows the HPLC profiles of guava, sweet potato, lemon, banana, toona, longan, wax apple, mango, caimito, rambutan, and cocoa, respectively. The solvent system identified Chl from 10 plant species within 30 min with a flow rate at 1 mL/min and detection at 667 nm. Chlide in ethanol crude extracts was detected within 10 min at 667 nm (Figure 1). According to the retention time, standards, and UV spectra, the peaks in Figure 1 were identified as Chl and chlide.

### 3.3. Cytotoxic Effect and Selectivity of Chlase-Treated Crude Extracts in the MTT Assay

**Figure 2:**
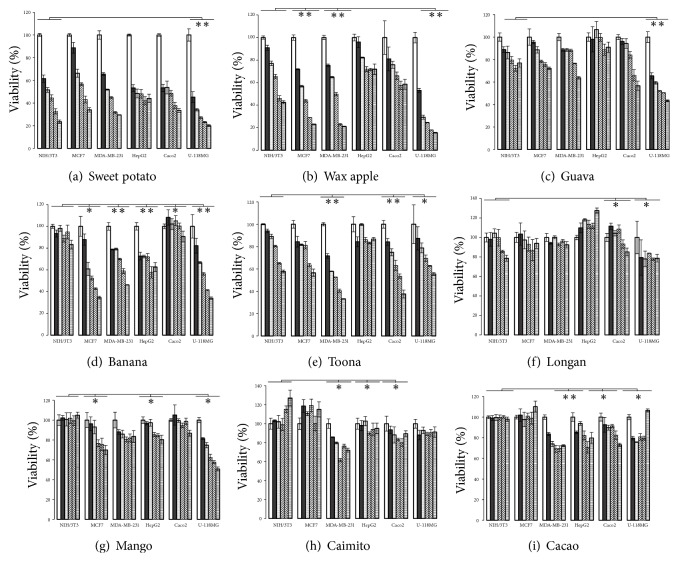
*Cytotoxicity of chlase-treated ethanol crude extracts on NIH/3T3, MCF7, MDA-MB-231, Hep G2, Caco2, and U-118MG cells determined by MTT assays*. The cells (5 × 10^4^/well) were stimulated with different doses of chlide (50, 80, 100, 150, and 200 *μ*g/mL). The bars represent the percentage growth inhibition of cells. All measurements made in 96-well plates were carried out using five technical replicates. When compared with the control, the results were statistically significant (*∗*P<0.05, *∗∗*P<0.01, and *∗∗∗*P<0.001).

In the present study, the cytotoxic effects of 10 chlase-treated crude extracts at a concentration range of 50–200 *μ*g/mL against human fibroblast cells (NIH/3T3), human breast cancer cell lines (MC7 and MDA-MB-231), hepatocellular carcinoma cells (Hep G2), colorectal adenocarcinoma cells (Caco2), and glioblastoma cells (U-118 MG) were determined by MTT assay (Figure 2). Chlase-treated crude extracts from guava induced the death of U-118 MG cells in a concentration-dependent manner with an IC_50_ value of 134 *μ*g/mL (P < 0.01), while MCF-7, MDA-MB-231, and Caco2 cells displayed moderate viability in response to guava (IC_50_ > 200 *μ*g/mL). For sweet potato, chlase-treated crude extracts induced a concentration-dependent cytotoxic response in all human cell lines tested, including NIH/3T3 cells (Figure 2(a)). Compared with the other plants, sweet potato presented a lower IC_50_ value, at 82.08, 122.29, 82.9, 63.73, 80.73, and 43.17 *μ*g/mL in NIH/3T3, MCF-7, MDA-MB-231, Hep G2, Caco2, and U-118 MG cells, respectively (Figure 2(b)). Chlase-treated crude extracts from banana presented high levels of cytotoxicity against all tested cell lines, especially MDA-MB-231, Hep G2, and U-118 MG cells (Figure 2(c)). The cytotoxic effect of chlase-treated crude extracts from toona was similar to that of sweet potato, with a slightly higher IC_50_ value, except for Hep G2 cells (Figure 2(d)). With longan, the greatest cytotoxicity was found in Hep G2, Caco2, and U-118 MG cell lines, and no evident effects were found in MCF7 and MDA-MB-231 cells (Figure 2(e)). For wax apple, significant and dose-dependent cytotoxicity was observed in MCF7, MDA-MB-231, and U-118 MG cells (Figure 2(f)). In NIH/3T3 cells, mango, caimito, and cacao presented no evidence of cytotoxicity. However, only small difference in chlase-treated crude extracts were observed between the effects of mango, caimito, and cacao in MCF7, MDA-MB-231, Hep G2, and U-118 MG cells, with an IC_50_ >200 *μ*g/mL (Figures 2(g), 2(h), and 2(i)).

Based on the dose-response curve, the IC_50_ of each chlase-treated crude extract was calculated, and these are summarized in Table 2. MCF7 cells were more sensitive to chlase-treated crude extracts of wax apple, banana, and lemon with an IC_50_ of 88.87, 104.41, and 117.47 *μ*g/mL, respectively. MDA-MD-231 cells were most sensitive to sweet potato, lemon, and wax apple, with IC_50_ values of 82.9, 95.75, and 97.83 *μ*g/mL, respectively. In Hep G2 cell lines, sweet potato had the lowest IC_50_ at 63.73 *μ*g/mL, while those of other plants were nearly 200 *μ*g/mL. In Caco2 cells, the IC_50_ values of sweet potato and lemon were 80.73 and 105.77 *μ*g/mL, respectively. U-118 MG cells, which represent the most sensitive of the tested cell lines, were responsive to sweet potato, lemon, wax apple, banana, and guava, with IC_50_ values of 43.17, 50.15, 52.64, 119.59, and 133.55 *μ*g/mL, respectively.

Selectivity index (SI) is defined as the ratio between the IC_50_ of chlase-treated crude extract in cancerous and normal NIH/3T3 cells. A SI exceeding 2 was considered to indicate high selectivity. We calculated the SI values to verify the therapeutic potential of plant extracts. Banana had the highest SI value at 4.6, 4.02, 2.57, and 2.5 in MCF7, U-118 MG, MDA-MB-231, and Hep G2 cell lines, respectively. Wax apple, lemon, and guava had the highest selectivity, with SI values of 2.75, 2.49, and 2.37, respectively, in U-118 MG cell lines. Toona showed high selectivity towards MDA-MB-231 cell lines with an SI of 2.12. Among the tested crude extracts, sweet potato exhibited promising cytotoxicity with the lowest IC_50_ values (43.17–82.9 *μ*g/mL) in U-118 MG, Hep G2, Caco2, and MDA-MB-231 cells.

**Figure 3:**
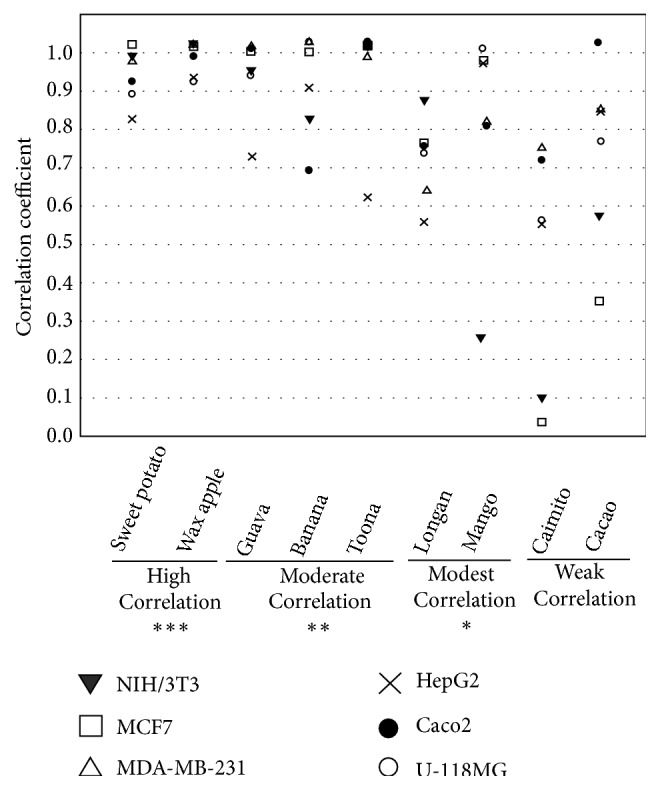
*Correlation Analyses*. IC50 values were evaluated by linear regression analysis. Correlation coefficients between cytotoxic activity and Chl/chlide contents were calculated by Pearson's correlation coefficient and showed here. High correlation: 0.7-0.99 (correlation coefficients); moderate correlation: 0.4-0.69; modest correlation: 0.1-0.39; weak correlation: 0.01-0.09.

Correlation coefficient in Figure 3 was calculated by Pearson's correlation coefficient. A negative correlation coefficient was observed for Chl/chlide content with cytotoxic activity and shown in red color (Figure 3 and Supplemental Table 1). We found that the correlation between Chl/chlide content and cytotoxic activity differs from plant to plant. According to the correlation coefficients, ten plants were divided into 4 groups. First, the high correlations (correlation coefficient: 0.7-0.99) were found in sweet potato, lemon and wax apple with P< 0.001. Guava, banana, and toona were classified into group 2 which was moderate (correlation coefficient: 0.4-0.69) with P< 0.01. Longan and mango belonged to group 3. In this group, the correlation was modest (correlation coefficient: 0.1-0.39) with P< 0.05. The weak correlations (correlation coefficient: 0.01-0.09) were observed in caimito and cacao (group 4).

**Figure 4:**
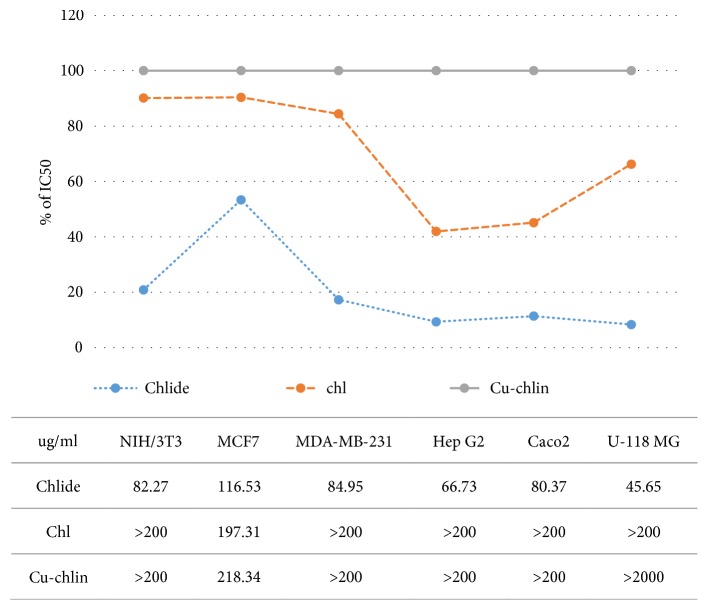
*IC*_*50*_ *of Chl and chlase-treated ethanol crude extracts from sweet potato in cancer cell lines*. IC_50_ values were calculated based on the results of the MTT assay and are shown in % of Cu-chlin. The IC_50_ values of different cell lines are shown at the bottom.

To confirm that chlide in chlase-treated crude extracts has an important effect on cell viability, the cytotoxicity of Chl and chlide in crude extracts from sweet potato and of Cu-chlin against MCF7, MDA-MD-231, Hep G2, Caco2, and U-118 MG cell lines was compared. Chl, chlide, and Cu-chlin were analyzed in an MTT assay at concentrations between 0 and 200 *μ*g/mL (Figure 4 and Supplement Figure 1). The results indicated that chlide in chlase-treated crude extracts from sweet potato exhibited promising cytotoxicity against MCF7, MDA-MD-231, Hep G2, Caco2, and U-118 MG cell lines, with IC_50_ values of 116.53, 84.95, 66.73, 80.37, and 45.65 *μ*g/mL, respectively. Chl possessed only moderate cytotoxicity against MCF7 cells, with an IC_50_ of 197.31 *μ*g/mL. Cu-chlin had low activity towards MCF7 cells, with an IC_50_ of 218.34 *μ*g/mL. These results were generally consistent with those observed in the screening test, confirming that U-118 MG, Hep G2, Caco2, and MDA-MB-231 cells were sensitive to chlase-treated crude extracts from sweet potato, for which the lowest IC_50_ values were found. Chl and Cu-chlin presented poor activity and selectivity compared with chlide.

### 3.4. Antioxidant Capacities of Chlide in Chlase-Treated Crude Extracts from Sweet Potato

**Figure 5:**
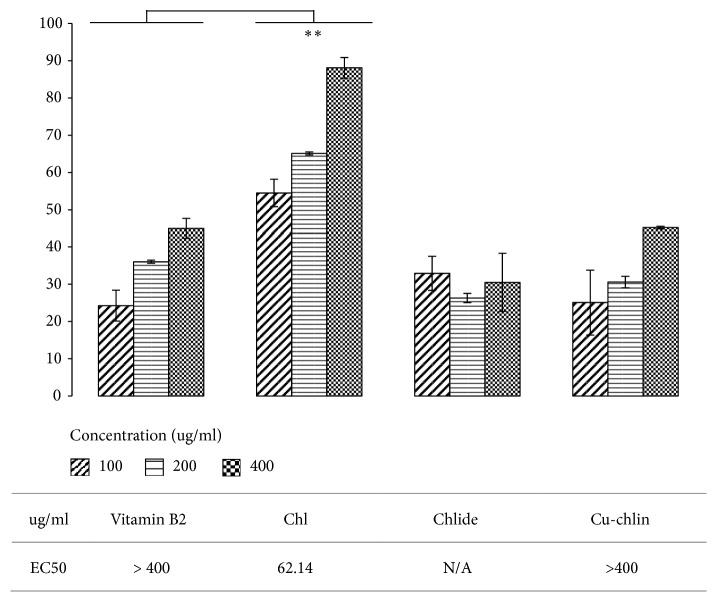
*DPPH assay*. DPPH radical scavenging activity was determined for Chl and chlide in sweet potato leaf ethanol extracts and Cu-chlin. The IC_50_ value of Chl, chlide, and Cu-chlin are shown at the bottom.

The antioxidant capacities of chl and chlide in chlase-treated crude extracts from sweet potato and Cu-chlin were compared by DPPH assay. The DPPH radical scavenging activity of Chl, Cu-chlin, and the positive control vitamin B2 increased in a dose-dependent manner (Figure 5). The scavenging rates of Chl reached 52.95, 65.11, and 88.62% at 100, 200, and 400 *μ*g/mL, respectively, which were higher than those observed for Vitamin B2. The scavenging rates of Cu-chlin were 25.68, 30.58, and 45.34%, respectively. The scavenging rate of chlide reached 31.01% at 100 *μ*g/mL. When the concentration increased to 200 *μ*g/mL, the scavenging activity of chlide (26.92%) was similar to that observed with 100 *μ*g/mL of vitamin B2 (24.2699%); this remained stable (26.09%) with 400 *μ*g/mL of chlide. The EC_50_ was calculated by SigmaPlot software and the result indicated that the EC_50_ values of vitamin B2 and Cu-chlin exceeded 400 *μ*g/mL, while that of Chl was 62.14 *μ*g/mL.

## 4. Discussion

Cancer is one of the devastating diseases to threaten human health worldwide. Their long history of use in traditional medicine make plants valuable tools for therapeutic purposes and for the treatment of cancer [63]. Since current therapeutic agents can cause systemic toxicity and cancer cell resistance, compounds from natural products represent promising alternative therapeutics for cancer management and treatment. Many clinically active anticancer agents are derived from plant source, including paclitaxel, etoposide, teniposide, vinblastine, vincristine, camptothecin, ingenol mebutate, omacetaxine, mepesuccinate, and combretastatin A4 phosphate [64].

Chl and its derivatives belong to a family of phytochemical plant pigments, which have been associated with cancer prevention [65]. Many studies have investigated natural Chl from leaf extracts and commercial copper chlorophyllin for their biological activities [66, 67]. In this study, MTT assay showed that chlase-treated crude extracts from banana were highly selective and significantly inhibited the growth of MCF7, MDA-MD-231, HepG2, and U-118 MG cell lines (Figure 2 and Table 2). Arun* et al*. (2018) found that the inflorescence of banana could induce the death of human colon cancer cells [68]. Harsha* et al*. (2017) also showed that ethyl acetate extracts of banana root exhibited high cytotoxicity (IC_50_ 60 *μ*g/mL) in MDA-MB-231 cells, inhibiting cell proliferation by up to 81% [69]. The IC_50_ values of chlide in MDA-MB-231 and MCF7 cells were slightly different at 84.95 and 116.53 *μ*g/mL, respectively. In MDA-MB-231 cells, the IC_50_ values of Chl and Cu-chlin were three-times higher than that of chlide. This finding is similar to that of a previous study that demonstrated that MDA-MB-231 cells were sensitive, while MCF7 cells were resistant to curcumin; this may be related to the SKP2-Cip/Kips signaling pathway [70]. Therefore, the differential susceptibilities of MCF7 and MDA-MB-231 cells against the cytotoxic effects of chlide may be due to the heterogeneity of cancer. It is reasonable to suggest that chlide with improved cytotoxicity and enhanced selectivity may influence the design of novel treatments for this tumour type.

Glioblastoma is a highly aggressive cancer with characteristics of common intracranial malignancy; it is highly lethal, resistant to available treatments, and associated with a poor prognosis. Current treatment options include combinations of surgery, radiotherapy, and chemotherapy with oral temozolomide [71]. The human colorectal adenocarcinoma Caco2 cell line is heterogeneous and frequently used as a model of the intestinal epithelial barrier [72]. In the present study, our results showed that U-118 MG was the most sensitive cell line, while Caco2 cells were the most resistant to the chlase-treated crude extracts tested. Initial screening results showed that extracts from 10 plant species (including guava, sweet potato, lemon, banana, and wax apple) presented promising activity against U-118 MG cells. Among these, only three (sweet potato, lemon, and wax apple) displayed potential cytotoxicity with an IC_50_ close to 50 *μ*g/mL. The potency of the extracts in descending order was as follows: banana, sweet potato, wax apple, and lemon. In 2015, Abusamra* et al*. found that the crude extract of* Onopordum acanthium* leaves presented high cytotoxicity through apoptosis in glioblastoma cells [73]. The use of methamphetamine as a coadjuvant was able to improve the delivery of doxorubicin and methotrexate to the brain, thus improving their cytotoxicity [73]. Moreover, Madi et al. (2016) showed that leaf extracts from* Moringa oleifera* decreased the viability of Hep G2, Caco2, Jurkat, and HEK293 cells [74]. A plant-derived compound, cycloart-24-ene-26-ol-3-one, isolated from the hexane extracts of* Aglaia exima* leaves decreased the viability of HT-29 and Caco2 cells in a dose- and time-dependent manner [75]. Two compounds (*β*-amyrin and *β*-sitosterol-3-O-glucoside) extracted from the leaves of* Prunus africana* had significant cytotoxicity towards Caco2 cells and low cytotoxicity in Hep G2 cells [76]. Therefore, chlide in chlase-treated ethanol extracts, a more hydrophilic derivative, may be able to cross the blood-brain barrier with effective cytotoxicity.

The use of a C18 column is suitable for the separation of Chl and its derivatives [77]. In Figure 1, HPLC profiles of Chl and chlide presented a typical UV-visible spectrum for each compound. Chl was detected at 667 nm after about 30 mins and chlide after 10 mins. However, the HPLC profile of lemon leaf exhibited a different pattern, which was detected at 10 and 5 mins for Chl and chlide, respectively. More analytical methods are needed to clarify the difference.

The antioxidant potential of Chl derivatives was determined in the DPPH radical scavenging assay based upon their hydrogen donating ability. In the present study, we show that at lower concentrations (100 *μ*g/mL), chlide, Cu-chlin, and vitamin B2 possess almost similar DPPH radical scavenging activity. As the concentration increased, Chl seemed to present a logarithmic increase in percentage inhibition over the concentration range used, achieving a maximum inhibition of about 90% and an IC_50_ value of about 62.14 *μ*g/mL, as shown in Figure 5. Neither chlide nor Cu-chlin possessed antioxidative activity at 400 *μ*g/mL, while Chl presented higher antioxidant activity than vitamin B2. Conversely, increasing the concentration of chlide to 400 *μ*g/mL decreased the percentage inhibition. Beyond this value, the percentage of inhibition appeared to approach a plateau at 32% with corresponding IC_50_ of 68 *μ*g/mL. These observations were consistent with similar findings that high Chl content in crude extracts had antioxidant and protective effects [16]. Therefore, in chlase-treated crude extracts of leaves, unknown components may cooperate with chlide and play a major role in its antioxidant effects.

Among the 10 plants studied, our results suggested that chlase-treated crude extracts from sweet potato were most effective against the five cancer cell lines tested (Figure 2 and Table 2). Chl/chlide content in sweet potato is highly correlated to its respective cytotoxic activity (Figure 4). The specificity of sweet potato extracts (SI) was low, suggesting that combination therapy with chlide in chlase-treated crude extracts may prove more efficacious. To compare the cytotoxic effects of chlide and Chl from sweet potato with that of commercial Cu-chlin, an MTT assay was performed. Cell growth was inhibited in the following descending order: U-118 MG, Hep G2, Caco2, MDA-MB-231, and MCF7 cells. Notably, the IC_50_ value of U-118 MG cells was 45.65 *μ*g/mL, while that of Chl and Cu-chlin exceeded 200 *μ*g/mL (Figure 4). This result further demonstrated that chlide from crude extracts was prone to have lower cytotoxic effect in cell lines tested. However, Kuete* et al*. reported that IC50 of a purified cytotoxic compound is 4 *μ*g/mL or 10 uM [78]. Considering this, chlide from leaves crude extracts could function as lead molecules or precursors to synthesize chlide derivatives with improved activity. Clinical significance from our data suggested that combination therapy with chemotherapeutics and chlide in crude extracts may prove more efficacious. Chlide may be used as a component of chemopreventive measures to attenuate important side effects induced by chemotherapeutics.

In the present study, the cytotoxicities of chlase-treated crude extracts from 10 plants were compared, revealing different ethanol crude extracts in the responsiveness and sensitivity of different cancer cells. Differences in the yield of Chl and chlide between ours and previous studies were likely due to different solvents, different methods of drying samples, and different harvested regions. The extraction method used in our study was not able to exclude hydrophilic compounds, such as anthocyanin, flavonoids, and carotenoids, indicated that other components may coextracted with Chl by ethanol. Therefore, the association of Chl/chlide content and its respective cytotoxic activity differs from plants to plants. In the group with high correlation, chlide may play an important role in its cytotoxic effect. This new finding from our data indicated that chlide of chlase-treated crude extract has different effects due to its other functional components, whether function as antagonist or agonist of chlide should be further studied.

Taken together, the lowest weight of chlase-treated crude extracts, the lowest IC50 values (43.17–82.9 *μ*g/mL) in U-118 MG, Hep G2, Caco2, and MDA-MB-231 cells, and a negative significant correlation coefficient were observed in sweet potato. Chlide in chlase-treated crude extracts from sweet potato may contribute to cytotoxic effect and then lead to its health benefits and recreational and medicinal purposes.

## 5. Conclusions

To the best of our knowledge, the cytotoxicity of chlide in plant leaf crude extracts has not previously been studied. In fact, most relevant studies carried out to date have investigated sodium copper chlorophyllin or extracts containing phytol and not the natural derivative (chlide) itself. The current study was the first of its kind to demonstrate that chlide in ethanol crude extracts from leaves has the potential to exert cytotoxic effects on cancer cell lines. It is reasonable to suggest that chlide itself, with improved cytotoxicity and enhanced selectivity, may drive the development of novel strategies for cancer treatment.

## Figures and Tables

**Table 1:** The concentration of Chl a and Chl b extracted from leaves of plants.

Plant species		Chl a (mg/ gDw)	Chl b (mg/ gDw)	CE (mg/gDw)
Guava	*Psidium guajava*	5.219	1.493	124.39
Sweet potato	*Ipomoea batatas*	3.481	0.996	43.175
Banana	*Musa paradisiaca*	2.921	1.031	47.76
Toona	*Toona sinensis*	9.800	5.419	148.19
Longan	*Dimocarpus longan*	7.044	1.903	183.15
Wax apple	*Syzygium samarangense*	5.423	1.955	94.29
Mango	*Mangifera indica*	8.407	2.599	291.77
Caimito	*Pouteria Caimito *	5.218	1.493	397.62
Cacao	*Theobroma cacao*	6.718	4.485	412.65

gDw: gram dry weight.

CE: chlase-treated crude extract contained chlide a/b.

**Table 2:** IC50 and SI values of chlase-treated crude extracts from leaves on cancer cell line.

Plant species	*MCF7*		*MDA-MB-231*		*Hep G2*		*Caco2*		*U-118 MG*		*NIH/3T3*
	IC50	SI	IC50	SI	IC50	SI	IC50	SI	IC50	SI	IC50
Guava	>200	1.03	>200	1.10	>200	0.72	>200	1.41	133.55	2.37	>200
Sweet potato	122.29	0.67	82.90	0.99	63.73	1.29	80.73	1.02	43.17	1.915	82.08
Lemon	117.47	1.06	95.75	1.31	>200	0.37	105.77	1.18	50.15	2.49	125.01
Banana	104.41	4.60	186.99	2.57	192.07	2.50	N/A	1.00	119.59	4.02	>200
Toona	>200	1.02	107.24	2.12	>200	0.74	154.63	1.47	206.01	1.10	>200
Longan	>200	0.72	>200	0.56	>200	N/A	>200	0.99	>200	1.28	>200
Wax apple	88.87	1.63	97.83	1.48	>200	0.57	>200	0.66	52.64	2.75	144.90
Mango	>200	N/A	>200	N/A	>200	N/A	>200	N/A	>200	N/A	>200
Caimito	>200	N/A	>200	N/A	>200	N/A	>200	N/A	>200	N/A	>200
Cacao	>200	N/A	>200	N/A	>200	N/A	>200	N/A	>200	N/A	>200

Data are presented as the mean ± standard deviation following 5 replicative experiments. IC50, half maximal inhibitory concentration (*μ*g/ml); SI, selectivity index; N/A: not available.

## Data Availability

The data used to support the findings of this study are restricted by the Jei-Fu Shaw in order to protect patient privacy. Data are available from Jei-Fu Shaw (shawjf@isu.edu.tw) for researchers who meet the criteria for access to confidential data.

## Supplementary Materials

Supplementary Materials
* Cell Viability of Cu-Chlin, Chl, and Chlase-Treated Crude Extracts from Sweet Potato*. Cell viability was examined by the ability of the cells to cleave the tetrazolium salt MTT [3-(4,5-dimethylthiazol-2-yl)-2,5-diphenyl tetrazolium bromide] (Sigma Chem., St. Louis, MO) by the mitochondrial enzyme succinate dehydrogenase following a previously described procedure [57]. Cells were incubated at the temperature used to acclimatize cell lines. The background absorbance of the culture medium was subtracted from the measured absorbance. Cells (5×10^4^/well) were stimulated with different doses of crude extracts (0, 100, and 200 *μ*g/mL). At the end of the incubation period, 24 h after stimulation, 20 *μ*L of the MTT solution was added per well. After treatment for 24 h, supernatants were removed from the wells and 1% MTT solution was added to each well. The plates were incubated for 4 h at 37°C and the optical density was determined at 595 nm using a multiwell spectrophotometer (Multiskan, Thermo Fisher Scientific, Waltham, MA). All measurements made in the 96-well plates were performed using five technical replicates. In addition, cell viability was examined microscopically for the presence of cytopathic effects. The half-maximal inhibitory concentration (IC50) was defined as the concentration required to inhibit cell viability by 50%. The IC50 value and the standard error of the mean (SEM) were calculated using a nonlinear regression curve contained in the SigmaPlot™ statistical software.* Statistical Analysis. *IC50 values of chlase-treated crude extracts from leaves were evaluated by linear regression analysis. Correlation coefficients between Chl/chlide content and cytotoxic activity were calculated by Pearson's correlation coefficient. The values were between +1 (black color) and −1 (red color). The absolute value of correlation coefficient ranges from 0.7 to 0.99, from 0.4 to 0.69, from 0.1 to 0.39, and from 0.01 to 0.09 which was defined as high, moderate, modest, and weak correlations. Values in Tables 1 and 2 were evaluated by linear regression analysis, and correlation coefficients were calculated by Pearson's correlation coefficient (CC). The values were between +1 (black color) and −1 (red color). High correlation: 0.7-0.99 (correlation coefficients); moderate correlation: 0.4-0.69; modest correlation: 0.1-0.39; weak correlation: 0.01-0.09.

## References

[1] Newman D. J., Cragg G. M. (2016). Natural products as sources of new drugs from 1981 to 2014. *Journal of Natural Products*.

[2] Wahab S. M., Jantan I., Haque M. A., Arshad L. (2018). Exploring the leaves of Annona muricata L. as a source of potential anti-inflammatory and anticancer agents. *Frontiers in Pharmacology*.

[3] Díaz-de-Cerio E., Verardo V., Gómez-Caravaca A., Fernández-Gutiérrez A., Segura-Carretero A. (2017). Health effects of psidium guajava l. leaves: an overview of the last decade. *International Journal of Molecular Sciences*.

[4] Marino S., Festa C., Zollo F. (2014). Antioxidant activity and chemical components as potential anticancer agents in the olive leaf (olea europaea l. cv leccino.) decoction. *Anti-Cancer Agents in Medicinal Chemistry*.

[5] Gawlik-Dziki U., Świeca M., Sułkowski M., Dziki D., Baraniak B., Czyz J. (2013). Antioxidant and anticancer activities of *Chenopodium quinoa* leaves extracts—in vitro study. *Food and Chemical Toxicology*.

[6] Ghasemzadeh A., Jaafar H. Z. (2013). Profiling of phenolic compounds and their antioxidant and anticancer activities in pandan (Pandanus amaryllifolius Roxb.) extracts from different locations of Malaysia. *BMC Complementary and Alternative Medicine*.

[7] Ghasemzadeh A., Jaafar H. Z. E., Karimi E., Rahmat A. (2014). Optimization of ultrasound-assisted extraction of flavonoid compounds and their pharmaceutical activity from curry leaf (Murraya koenigii L.) using response surface methodology. *BMC Complementary and Alternative Medicine*.

[8] Hseu Y. C., Chen S. C., Lin W. H. (2011). Toona sinensis (leaf extracts) inhibit vascular endothelial growth factor (VEGF)-induced angiogenesis in vascular endothelial cells. *Journal of Ethnopharmacology*.

[9] Pornprasertpol A., Sereemaspun A., Sooklert K., Satirapipatkul C., Sukrong S. (2015). Anticancer activity of selected colocasia gigantia fractions. *Journal of the Medical Association of Thailand*.

[10] Wang D. S., Rizwani G. H., Guo H. (2014). Annona squamosa Linn: cytotoxic activity found in leaf extract against human tumor cell lines. *Pakistan Journal of Pharmaceutical Sciences*.

[11] Borrelli F., Pagano E., Romano B. (2014). Colon carcinogenesis is inhibited by the TRPM8 antagonist cannabigerol, a Cannabis-derived non-psychotropic cannabinoid. *Carcinogenesis*.

[12] Romano B., Borrelli F., Pagano E., Cascio M. G., Pertwee R. G., Izzo A. A. (2014). Inhibition of colon carcinogenesis by a standardized *Cannabis sativa* extract with high content of cannabidiol. *Phytomedicine*.

[13] Romano B., Fasolino I., Pagano E. (2014). The chemopreventive action of bromelain, from pineapple stem ( *Ananas comosus* L.), on colon carcinogenesis is related to antiproliferative and proapoptotic effects. *Molecular Nutrition & Food Research*.

[14] Cho M., Park G., Kim S., Amna T., Lee S., Shin W. (2014). Glioblastoma-specific anticancer activity of pheophorbide a from the edible red seaweed grateloupia elliptica. *Journal of Microbiology and Biotechnology*.

[15] Cieckiewicz E., Angenot L., Gras T., Kiss R., Frédérich M. (2012). Potential anticancer activity of young Carpinus betulus leaves. *Phytomedicine*.

[16] El-Sayed W. M., Hussin W. A., Mahmoud A. A., AlFredan M. A. (2013). The Conyza triloba extracts with high chlorophyll content and free radical scavenging activity had anticancer activity in cell lines. *BioMed Research International*.

[17] Rai V., Tandon P. K., Khatoon S. (2014). Effect of chromium on antioxidant potential of catharanthus roseus varieties and production of their anticancer alkaloids: vincristine and vinblastine. *BioMed Research International*.

[18] Jantan I., Ahmad W., Bukhari S. N. (2018). Corrigendum: Plant-derived immunomodulators: an insight on their preclinical evaluation and clinical trials. *Frontiers in Plant Science*.

[19] Arshad L., Jantan I., Bukhari S. N., Haque M. A. (2017). Immunosuppressive effects of natural *α*,*β*-unsaturated carbonyl-based compounds, and their analogs and derivatives, on immune cells: a review. *Frontiers in Pharmacology*.

[20] Haque M. A., Jantan I., Abbas Bukhari S. N. (2017). Tinospora species: An overview of their modulating effects on the immune system. *Journal of Ethnopharmacology*.

[21] Haque M. A., Jantan I., Arshad L., Bukhari S. N. A. (2017). Exploring the immunomodulatory and anticancer properties of zerumbone. *Food & Function*.

[22] Mohamed S. I., Jantan I., Haque M. A. (2017). Naturally occurring immunomodulators with antitumor activity: An insight on their mechanisms of action. *International Immunopharmacology*.

[23] McQuistan T. J., Simonich M. T., Pratt M. M. (2012). Cancer chemoprevention by dietary chlorophylls: A 12,000-animal dose–dose matrix biomarker and tumor study. *Food and Chemical Toxicology*.

[24] Weemaes C. A., Ooms V., Van Loey A. M., Hendrickx M. E. (1999). Kinetics of chlorophyll degradation and color loss in heated broccoli juice. *Journal of Agricultural and Food Chemistry*.

[25] Hörtensteiner S., Kräutler B. (2011). Chlorophyll breakdown in higher plants. *Biochimica et Biophysica Acta*.

[26] Kuai B., Chen J., Hörtensteiner S. (2018). The biochemistry and molecular biology of chlorophyll breakdown. *Journal of Experimental Botany*.

[27] Kräutler B. (2016). Breakdown of chlorophyll in higher plants-phyllobilins as abundant, yet hardly visible signs of ripening, senescence, and cell death. *Angewandte Chemie International Edition*.

[28] Chiu L. C., Kong C. K., Ooi V. E. (2005). The chlorophyllin-induced cell cycle arrest and apoptosis in human breast cancer MCF-7 cells is associated with ERK deactivation and Cyclin D1 depletion. *International Journal of Molecular Medicine*.

[29] Díaz G. D., Li Q., Dashwood R. H. (2003). Caspase-8 and apoptosis-inducing factor mediate a cytochrome c-independent pathway of apoptosis in human colon cancer cells induced by the dietary phytochemical chlorophyllin. *Cancer Research*.

[30] Chimploy K., Díaz G. D., Li Q. (2009). E2F4 and ribonucleotide reductase mediate S‐phase arrest in colon cancer cells treated with chlorophyllin. *International Journal of Cancer*.

[31] Hsu C., Yang C., Chen C., Chao P., Hu S. (2005). Effects of chlorophyll-related compounds on hydrogen peroxide induced dna damage within human lymphocytes. *Journal of Agricultural and Food Chemistry*.

[32] Hsu C., Chen Y., Chao P., Chen C., Hsieh L., Hu S. (2008). Naturally occurring chlorophyll derivatives inhibit aflatoxin B1-DNA adduct formation in hepatoma cells. *Mutation Research - Genetic Toxicology and Environmental Mutagenesis*.

[33] Guo H., Pan X., Mao R. (2011). Alkylated porphyrins have broad antiviral activity against hepadnaviruses, flaviviruses, filoviruses, and arenaviruses. *Antimicrobial Agents and Chemotherapy*.

[34] Komiya T., Kyohkon M., Ohwaki S. (1999). Phytol induces programmed cell death in human lymphoid leukemia Molt 4B cells. *International Journal of Molecular Medicine*.

[35] Hibasami H., Kyohkon M., Ohwaki S. (2000). Pheophorbide a, a moiety of chlorophyll a, induces apoptosis in human lymphoid leukemia molt 4B cells. *International Journal of Molecular Medicine*.

[36] Silva R. O., Sousa F. B., Damasceno S. R. (2014). Phytol, a diterpene alcohol, inhibits the inflammatory response by reducing cytokine production and oxidative stress. *Fundamental Clinical Pharmacology*.

[37] Tang P. M.-K., Chan J. Y.-W., Au S. W.-N. (2006). Pheophorbide a, an active compound isolated from *Scutellaria barbata*, possesses photodynamic activities by inducing apoptosis in human hepatocellular carcinoma. *Cancer Biology & Therapy*.

[38] Lee W. Y., Lim D. S., Ko S. H. (2004). Photoactivation of pheophorbide a induces a mitochondrial-mediated apoptosis in Jurkat leukaemia cells. *Journal of Photochemistry and Photobiology B: Biology*.

[39] Chan J., Tang P., Hon P. (2006). Pheophorbide a, a major antitumor component purified from scutellaria barbata, induces apoptosis in human hepatocellular carcinoma cells. *Planta Medica*.

[40] Barrett J. R. (2002). Cancer. plants provide prevention. *Environmental Health Perspectives*.

[41] Hirose M., Nishikawa A., Shibutani M., Imai T., Shirai T. (2002). Chemoprevention of heterocyclic amine-induced mammary carcinogenesis in rats. *Environmental and Molecular Mutagenesis*.

[42] Kelloff G. J., Lippman S. M., Dannenberg A. J. (2006). Progress in chemoprevention drug development: The promise of molecular biomarkers for prevention of intraepithelial neoplasia and cancer - A plan to move forward. *Clinical Cancer Research*.

[43] Williams D. E. (2012). The rainbow trout liver cancer model: Response to environmental chemicals and studies on promotion and chemoprevention. *Comparative Biochemistry and Physiology Part C: Toxicology & Pharmacology*.

[44] Wogan G. N., Kensler T. W., Groopman J. D. (2012). Present and future directions of translational research on aflatoxin and hepatocellular carcinoma. A review. *Food Additives & Contaminants: Part A*.

[45] Ferguson L. R., Philpott M., Karunasinghe N. (2004). Dietary cancer and prevention using antimutagens. *Toxicology*.

[46] Ding X.-W., Ding X.-L., Zheng S., Yang H.-J. (2004). CHL prevent colon neoplasms in mice and its selective inhibition on COX-2. *Ai Zheng*.

[47] Kikuchi A., Yamamoto H., Sato A., Matsumoto S. (2011). New insights into the mechanism of Wnt signaling pathway activation. *International Review of Cell and Molecular Biology*.

[48] Chou Y.-L., Ko C.-Y., Chen L.-F. O., Yen C.-C., Shaw J.-F. (2015). Purification and immobilization of the recombinant Brassica oleracea Chlorophyllase 1 (BoCLH1) on DIAION(R)CR11 as potential biocatalyst for the production of chlorophyllide and phytol. *Molecules*.

[49] Chou Y., Ko C., Yen C., Chen L. O., Shaw J. (2015). A Novel Recombinant Chlorophyllase1 from *Chlamydomonas reinhardtii* for the Production of Chlorophyllide Derivatives. *Journal of Agricultural and Food Chemistry*.

[50] Chou Y., Lee Y., Yen C., Chen L. O., Lee L., Shaw J. (2016). A novel recombinant chlorophyllase from cyanobacterium *Cyanothece sp* . ATCC 51142 for the production of bacteriochlorophyllide a. *Biotechnology and Applied Biochemistry*.

[51] Yen C., Chuang Y., Ko C. (2016). Immobilization of chlamydomonas reinhardtii clh1 on aptes-coated magnetic iron oxide nanoparticles and its potential in the production of chlorophyll derivatives. *Molecules*.

[52] Knudson L. L., Tibbitts T. W., Edwards G. E. (1977). Measurement of Ozone Injury by Determination of Leaf Chlorophyll Concentration. *Plant Physiology*.

[53] Wintermans J. F., De Mots A. (1965). Spectrophotometric characteristics of chlorophylls a and b and their phenophytins in ethanol. *Biochimica et biophysica acta*.

[54] Qin H., Li S., Li D. (2013). An improved method for determining phytoplankton chlorophyll a concentration without filtration. *Hydrobiologia*.

[55] Pružinská A., Tanner G., Aubry S. (2005). Chlorophyll breakdown in senescent Arabidopsis leaves: characterization of chlorophyll catabolites and of chlorophyll catabolic enzymes involved in the degreening reaction. *Plant Physiology*.

[56] Van Heukelem L., Thomas C. S. (2001). Computer-assisted high-performance liquid chromatography method development with applications to the isolation and analysis of phytoplankton pigments. *Journal of Chromatography A*.

[57] Schelbert S., Aubry S., Burla B. (2009). Pheophytin Pheophorbide Hydrolase (Pheophytinase) Is Involved in Chlorophyll Breakdown during Leaf Senescence in *Arabidopsis*. *The Plant Cell*.

[58] Kumar P., Nagarajan A., Uchil P. D. (2018). Analysis of cell viability by the mtt assay. *Cold Spring Harbor Protocols*.

[59] Koh R. Y., Lim F. P., Ling L. S. (2017). Anticancer mechanisms of Strobilanthes crispa Blume hexane extract on liver and breast cancer cell lines. *Oncology Letters*.

[60] Mahavorasirikul W., Viyanant V., Chaijaroenkul W., Itharat A., Na-Bangchang K. (2010). Cytotoxic activity of Thai medicinal plants against human cholangiocarcinoma, laryngeal and hepatocarcinoma cells *in vitro*. *BMC Complementary and Alternative Medicine*.

[61] Yuan C., Du L., Jin Z., Xu X. (2013). Storage stability and antioxidant activity of complex of astaxanthin with hydroxypropyl-*β*-cyclodextrin. *Carbohydrate Polymers*.

[62] Chen Z., Bertin R., Froldi G. (2013). EC50 estimation of antioxidant activity in DPPH assay using several statistical programs. *Food Chemistry*.

[63] Kephart J. C. (1955). Chlorophyll derivatives—Their chemistry? commercial preparation and uses. *Economic Botany*.

[64] Newman D. J., Cragg G. M., Kingston D. G. I., Wermuth C. G., Aldous D., Raboisson P., Rognan D. (2015). Natural products as pharmaceuticals and sources for lead structures∗∗note: this chapter reflects the opinions of the authors. *The Practice of Medicinal Chemistry*.

[65] Fahey J. W., Stephenson K. K., Dinkova-Kostova A. T., Egner P. A., Kensler T. W., Talalay P. (2005). Chlorophyll, chlorophyllin and related tetrapyrroles are significant inducers of mammalian phase 2 cytoprotective genes. *Carcinogenesis*.

[66] Ferruzzi M. G., Blakeslee J. (2007). Digestion, absorption, and cancer preventative activity of dietary chlorophyll derivatives. *Nutrition Research*.

[67] Nagini S., Palitti F., Natarajan A. T. (2015). Chemopreventive potential of chlorophyllin: A review of the mechanisms of action and molecular targets. *Nutrition and Cancer*.

[68] Arun K. B., Madhavan A., Reshmitha T. R., Thomas S., Nisha P. (2018). Musa paradisiaca inflorescence induces human colon cancer cell death by modulating cascades of transcriptional events. *Food & Function*.

[69] Raj M. H., Ghosh D., Banerjee R., Salimath B. P. (2016). Suppression of VEGF-induced angiogenesis and tumor growth by eugenia jambolana, musa paradisiaca, and coccinia indica extracts. *Pharmaceutical Biology*.

[70] Jia T., Zhang L., Duan Y. (2014). The differential susceptibilities of MCF-7 and MDA-MB-231 cells to the cytotoxic effects of curcumin are associated with the PI3K/Akt-SKP2- Cip/Kips pathway. *Cancer Cell International*.

[71] Messaoudi K., Clavreul A., Lagarce F. (2015). Toward an effective strategy in glioblastoma treatment. Part I: resistance mechanisms and strategies to overcome resistance of glioblastoma to temozolomide. *Drug Discovery Therapy*.

[72] Lea T., Verhoeckx K., Cotter P., Lopez-Exposito I. (2015). Caco-2 cell line. *The Impact of Food Bioactives on Health: in Vitro and Ex Vivo Models*.

[73] Abusamra Y. A.-K., Scuruchi M., Habibatni S. (2015). Evaluation of putative cytotoxic activity of crude extracts from Onopordum acanthium leaves and Spartium junceum flowers against the U-373 glioblastoma cell line. *Pakistan Journal of Pharmaceutical Sciences*.

[74] Madi N., Dany M., Abdoun S., Usta J. (2016). Moringa oleifera's nutritious aqueous leaf extract has anticancerous effects by compromising mitochondrial viability in an ros-dependent manner. *Journal of the American College of Nutrition*.

[75] Leong K. H., Looi C. Y., Loong X. M. (2016). Cycloart-24-ene-26-ol-3-one, a new cycloartane isolated from leaves of *Aglaia exima* triggers tumour necrosis factor-receptor 1-mediated caspase-dependent apoptosis in colon cancer cell line. *PLoS ONE*.

[76] Maiyo F., Moodley R., Singh M. (2016). Phytochemistry, cytotoxicity and apoptosis studies of *β*-sitosterol-3-oglucoside and *β* -amyrin from prunus africana. *Africa Journal of Traditional Complementary and Alternative Medicine*.

[77] Huang S., Hung C., Wu W., Chen B. (2008). Determination of chlorophylls and their derivatives in Gynostemma pentaphyllum Makino by liquid chromatography–mass spectrometry. *Journal of Pharmaceutical and Biomedical Analysis*.

[78] Kuete V., Ango P. Y., Yeboah S. O. (2014). Cytotoxicity of four aframomum species (a. arundinaceum, a. alboviolaceum, a. kayserianum and a. polyanthum) towards multi-factorial drug resistant cancer cell lines. *BMC Complementary and Alternative Medicine*.

