# Supplementary material for: Cytotoxic Effects of Chlorophyllides in Ethanol Crude Extracts from Plant Leaves
Source: Evid Based Complement Alternat Med. 2019 Jul 15;2019:9494328. doi: 10.1155/2019/9494328 (PMC6662445; doi:10.1155/2019/9494328)
Supplement: Supplementary Materials — Cell Viability of Cu-Chlin, Chl, and Chlase-Treated Crude Extracts from Sweet Potato. Cell viability was examined by the ability of the cells to cleave the tetrazolium salt MTT [3-(4,5-dimethylthiazol-2-yl)-2,5-diphenyl tetrazolium bromide] (Sigma Chem., St. Louis, MO) by the mitochondrial enzyme succinate dehydrogenase following a previously described procedure [57]. Cells were incubated at the temperature used to acclimatize cell lines. The background absorbance of the culture medium was subtracted from the measured absorbance. Cells (5×10^4^/well) were stimulated with different doses of crude extracts (0, 100, and 200 μg/mL). At the end of the incubation period, 24 h after stimulation, 20 μL of the MTT solution was added per well. After treatment for 24 h, supernatants were removed from the wells and 1% MTT solution was added to each well. The plates were incubated for 4 h at 37°C and the optical density was determined at 595 nm using a multiwell spectrophotometer (Multiskan, Thermo Fisher Scientific, Waltham, MA). All measurements made in the 96-well plates were performed using five technical replicates. In addition, cell viability was examined microscopically for the presence of cytopathic effects. The half-maximal inhibitory concentration (IC50) was defined as the concentration required to inhibit cell viability by 50%. The IC50 value and the standard error of the mean (SEM) were calculated using a nonlinear regression curve contained in the SigmaPlot™ statistical software. Statistical Analysis. IC50 values of chlase-treated crude extracts from leaves were evaluated by linear regression analysis. Correlation coefficients between Chl/chlide content and cytotoxic activity were calculated by Pearson's correlation coefficient. The values were between +1 (black color) and −1 (red color). The absolute value of correlation coefficient ranges from 0.7 to 0.99, from 0.4 to 0.69, from 0.1 to 0.39, and from 0.01 to 0.09 which was defined as high, moderate, modest, and weak [file 9494328.f1.docx]

**Supplement Fig. 1**

100

Concentration (ug/ml)

400

200

**Supplement Fig. 1 The effects of Chl and chlase-treated ethanol crude extracts from sweet potato on the growth of NIH/3T3, MCF7, MDA-MB-231, Hep G2, Caco2, and U-118MG cells was investigated using MTT assays**. Commercial Cu-chl was also tested in cancer cell lines. The cells (5 × 10^4^/well) were stimulated with different concentrations of chemicals (50, 80, 100, 150, and 200 μg/mL). Measurements in each 96-well plate were performed as five technical replicates. *P<0.05 and ***P<0.001, compared with the untreated control.

**Supplement Table 1 The values of correlation coefficient between MTT activity and chl/chlide contents.**

|  | Guava | Sweet potato | Lemon | Banana | Toona | Longan | Wax apple | Mango | Caimito | Cacao |
| --- | --- | --- | --- | --- | --- | --- | --- | --- | --- | --- |
| NIH/3T3 | (0.902) | (0.945) | (0.952) | (0.774) | (0.983) | (0.831) | (0.976) | (0.175) | (0.057) | (0.519) |
| MCF7 | (0.954) | (0.975) | (0.954) | (0.955) | (0.983) | (0.712) | (0.967) | (0.932) | 0.014 | (0.302) |
| MDA-MB-231 | (0.971) | (0.933) | (0.950) | (0.986) | (0.959) | (0.584) | (0.976) | (0.768) | (0.709) | (0.797) |
| Hep G2 | (0.666) | (0.770) | (0.890) | (0.859) | (0.572) | (0.497) | (0.878) | (0.922) | (0.497) | (0.789) |
| Caco2 | (0.961) | (0.876) | (0.847) | (0.634) | (0.995) | (0.701) | (0.937) | (0.758) | (0.676) | (0.975) |
| U-118 MG | (0.886) | (0.839) | (0.866) | (0.982) | (0.982) | (0.685) | (0.870) | (0.964) | (0.507) | (0.713) |

Values in Tables 1 and 2 were evaluated by linear regression analysis, and correlation coefficients was calculated by Pearson’s correlation coefficient (CC). The values were between +1 (black color) and −1 (red color).

Highly correlation: 0.7-0.99 (correlation coefficients); Moderately correlation: 0.4-0.69; Modestly correlation: 0.1-0.39; Weakly correlation: 0.01-0.09
